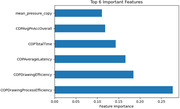# DCTclock metrics differentiate between amnestic and vascular mild cognitive impairment

**DOI:** 10.1002/alz.089745

**Published:** 2025-01-03

**Authors:** Brendan Haas, Ali Jannati, John Showalter, David Bates, Sean Tobyne, Alvaro Pascual‐Leone

**Affiliations:** ^1^ Linus Health, Boston, MA USA; ^2^ Harvard Medical School, Boston, MA USA; ^3^ Hebrew SeniorLife, Boston, MA USA

## Abstract

**Background:**

Amnestic and vascular dementia are two common types of dementia. Currently, diagnosing and differentiating between these two conditions requires comprehensive neuropsychological testing, neuroimaging studies, and cerebrospinal fluid analysis. Identifying these conditions at early stages, i.e., mild cognitive impairment (MCI), is crucial for maximizing the therapeutic benefits of pharmacological agents or lifestyle modifications. Here we evaluated the utility of metrics derived from the DCTclock, a brief ML‐enabled digital cognitive assessment, in differentiating between amnestic and vascular MCI.

**Method:**

We analyzed DCTclock tests from N = 215 participants (age 48‐90, 126 females, education = 6‐20 yrs) including patients at Lahey Hospital and Medical Center and participants in the Framingham Heart Study, for whom a diagnosis of vascular (n = 46) or amnestic (n = 169) MCI was available. Mann‐Whitney *U* Tests were used to compare the distributions for 78 features derived from the DCTclock performance. Random forest models with recursive feature elimination and 5‐fold cross‐validation were used to differentiate between the two MCI subtypes.

**Result:**

16 DCTclock features returned p‐values below the 0.05 threshold for statistical significance, and one feature reached the Bonferroni‐corrected threshold of 6.4e‐4. The features showing the largest difference between the two populations were the total time to complete the copy clock (p = 4.2e‐4), drawing process efficiency on the copy clock (p = 1.7e‐3), and average latency on the copy clock (p = 3.7e‐3). 13 of the 16 top features were derived from the copy clock test. A random forest model using only 6 features (figure below) returned an accuracy of 77.9%.

**Conclusion:**

Metrics derived from the process of DCTclock performance, especially those obtained during the copy clock condition, showed utility for differentiating between vascular and amnestic MCI. Comparing cognitive phenotypes at the early stage of MCI is arguably a more difficult task than comparing those phenotypes among individuals who have progressed to dementia and thus exhibit more pronounced signs of cognitive impairment. More data from individuals with such cognitive profiles are needed for building robust ML models that can differentiate among MCI subtypes with higher accuracy.